# Ectomycorrhizal Fungal Communities and Enzymatic Activities Vary across an Ecotone between a Forest and Field

**DOI:** 10.3390/jof1020185

**Published:** 2015-08-28

**Authors:** Megan A. Rúa, Becky Moore, Nicole Hergott, Lily Van, Colin R. Jackson, Jason D. Hoeksema

**Affiliations:** 1 Department of Biology, University of Mississippi, Oxford, MS 38677, USA; E-Mails: babrashe@gmail.com (B.M.); nhergott@vols.utk.edu (N.H.); lhvan@go.olemiss.edu (L.V.); cjackson@olemiss.edu (C.R.J.); hoeksema@olemiss.edu (J.D.H.); 2 Department of Ecology and Evolutionary Biology, University of Tennessee, Knoxville, TN 37966, USA

**Keywords:** β-glucosidase, carbon, nitrogen, peroxidase, phenol oxidase, phosphatase, *Pinus taeda*, soil texture

## Abstract

Extracellular enzymes degrade macromolecules into soluble substrates and are important for nutrient cycling in soils, where microorganisms, such as ectomycorrhizal (ECM) fungi, produce these enzymes to obtain nutrients. Ecotones between forests and fields represent intriguing arenas for examining the effect of the environment on ECM community structure and enzyme activity because tree maturity, ECM composition, and environmental variables may all be changing simultaneously. We studied the composition and enzymatic activity of ECM associated with loblolly pine (*Pinus taeda*) across an ecotone between a forest where *P. taeda* is established and an old field where *P. taeda* saplings had been growing for <5 years. ECM community and environmental characteristics influenced enzyme activity in the field, indicating that controls on enzyme activity may be intricately linked to the ECM community, but this was not true in the forest. Members of the Russulaceae were associated with increased phenol oxidase activity and decreased peroxidase activity in the field. Members of the Atheliaceae were particularly susceptible to changes in their abiotic environment, but this did not mediate differences in enzyme activity. These results emphasize the complex nature of factors that dictate the distribution of ECM and activity of their enzymes across a habitat boundary.

## 1. Introduction

Many early responses to global change have been investigated in ecotones, the transitional area between two ecosystem types [1,2]. These habitat boundaries are particularly sensitive indicators of environmental change [3,4] because they represent extremely heterogeneous landscapes and are prone to shifting towards one habitat or the other in response to environmental change. Ecotones are also characterized by high biological diversity because of the co-occurrence of species from adjacent habitats and operate as important controls for nutrient, sediment, and water dynamics [3]. As anthropogenic changes to temperature and nutrient dynamics create vegetation shifts, ecotone areas are increasing, establishing new habitats and species interactions.

Despite their importance, a complete understanding of the biotic and abiotic factors that shape these mosaic environments remains elusive. Our knowledge of species-interactions, such as those between vegetation and mycorrhizal fungi is particularly limited, as the species which occupy ecotones vary greatly with the bordering ecosystems. Because association with mycorrhizal fungi is ubiquitous for vegetation in most ecosystems [5], and mycorrhizal fungal communities typically shift along with plant communities during succession [6], understanding the distribution and function of mycorrhizal fungi is an important component for understanding these sensitive environments. Mycorrhizal fungi form symbiotic relationships with their host plant such that they receive carbon (C) in the form of carbohydrates from the plant in exchange for providing mineral nutrients, such as nitrogen (N) and phosphorus (P), acquired from the soil [7]. Ectomycorrhizal (ECM) fungi associate primarily with woody tree species and are an extremely diverse, containing more than 5000 described species from the fungal order Agaricales alone [8]. Mycorrhizal fungal species differ substantially in resource utilization, colonization, reproductive strategy, and response to disturbance [8,9]. These characteristics have contributed to the ability of ECM fungi to survive and thrive in a variety of environments and contribute to their potential importance in ecotones.

Some of the functional diversity of ECM fungi may be a result of their ability to produce extracellular enzymes that help them obtain nutrients, such as N and P, by degrading complex molecules in soil organic matter, including amino acids, proteins, cellulose, hemicellulose, lignin, and chitin [10,11,12,13]. ECM fungi can produce a large range of extracellular and cytosolic enzymes that break down N and P containing compounds [14,15,16,17], but the diversity of these enzymes in terms of both production and function is only now becoming clear. The hydrolytic enzymes β-glucosidase and phosphatase are important enzymes for the breakdown of cellulose (β-glucosidase), which makes up a large part of plant cell walls and releases C, or the degradation of amino acids, proteins, DNA, and other P-containing compounds (phosphatase; [16]). In contrast, the oxidative enzymes peroxidase and phenol oxidase are important in the oxidative degradation of lignin molecules (a major component of cell walls in woody plant tissue) and other complex polyphenolic compounds.

ECM fungi can vary in their production of extracellular enzymes [18], possibly as a result of environmental conditions. When rates of photosynthesis are diminished, available C from the plant is suppressed; in such contexts, it may be more advantageous for ECM fungi to acquire C directly from the soil rather than depending on its association with the host plant [16,18]. Alternatively, in situations where the host’s root mass is beginning to decline, such as when the C supply is low or a plant is senescing, it would be beneficial for the ECM fungus to start decomposition of its host’s root tissue sooner rather than later, allowing the fungus to escape the dying root and to use the nutrients contained within it to support extraradical mycelia during the search for alternative living and non-colonized roots [19]. Additionally, different species of ECM fungi may also vary in their ability to enzymatically obtain nutrients from different substrates in the soil, and this variation might explain their distribution among different ecological niches [20,21,22,23]. Consequently, some ECM fungal species may be selected for particular environments because of their enzymatic capabilities, but the plasticity of this enzymatic capability in different environments may also be important for other species.

The transition between an old field and a forest represents an intriguing location to explore the effect of differing environmental variables on not only mycorrhizal community structure but also on ECM fungal enzyme activities because this transition contains several changes that could impact the enzymatic activities of ECM fungi. Specifically, mature undisturbed forests often contain a deep organic litter layer of decaying leaves and wood, as well as a higher content of organic material in soils, which are not present in an open field. Thus, it might be expected that ECM fungi in forests would be producing larger amounts of oxidative enzymes (the ligninases) to degrade both lignin and its’ polyphenolic breakdown products. In open fields, where the litter layer may be more limited and contain much less woody debris, ligninases would be less beneficial, and ECM fungi may produce more enzymes related to nutrient acquisition via the degradation of less recalcitrant organic substrates, such as cellulose. Such variation in catabolic enzyme production may correlate with the ability of particular ECM fungi to sequester nutrients from the soil, functionally altering their ecological niche, and potentially leading to differences in ECM fungal community structure between forest and field habitats.

In addition to differences in the amount of leaf litter and woody material composing the rhizosphere [20,21], differences in soil properties between forest and field environments may also affect ECM species distribution and success. Lower concentrations of nutrients could affect the distribution of ECM species in the soil [24,25], changing the community composition of the fungi. Specifically, lower availability of N or P would favor ECM species that are readily able to break down and reabsorb N and P via the production of extracellular enzymes [24,25]. This hypothesis would predict that in the field, where the soils tended to exhibit lower N content, there could be selection for ECM species that are able to better mobilize P or N. Finally, ECM fungi have long been known to alter plant water relations [7] and are thought to be particularly important when water availability is limiting, further setting up the potential for a difference in ECM fungal community structure between the forest and field. Alternatively, individual ECM fungal species may exhibit plasticity in their enzymatic capability, which might also result in enzymatic patterns along the ecotone, leading to a more direct influence of environmental variables on patterns of enzymatic activity. Assessing the degree to which these patterns occur could help address whether there is an ecological impact of a change in ECM fungal community composition across an ecotone, such as through changes in ECM fungal enzymatic functionality.

In this study, we examine the impact of habitat on both the community structure and activity of ECM fungi in an ecotone where tree maturity, environmental conditions, and ECM composition may all be changing simultaneously. Specifically, we studied ECM fungal community structure and enzymatic activity associated with loblolly pine (*Pinus taeda*), within a transitional zone between a forest where *P. taeda* is a dominant member of the canopy to an old field where *P. taeda* saplings have been growing since the cessation of field management five years prior. *P. taeda* is an ecologically and economically important species in the Southeastern U.S. and accounts for 16% of harvested wood for industrial use in the U.S. and 5% worldwide [26]. It is also considered a major bioenergy feedstock [27]. The relationship between pine species and ECM colonization is particularly tight as the mutualistic relationship between ECM fungi and pines has been shown to be necessary for the growth and success of the trees [7], especially in the establishment of pines in systems undergoing succession [28]. The symbiotic association of pine species and ECM fungi has likely been reinforced evolutionarily because pines generally grow in sandy, nutrient poor soils [29], requiring them to attain much of the micronutrients in the soil via their ECM fungi. We assayed the activity of hydrolytic and oxidative enzymes involved in the degradation of cellulose, organic P, and lignin containing compounds by focusing on four classes of enzymes previously shown to be functionally important in ECM fungi: β-glucosidase, phosphatase, phenol oxidase, and peroxidase [15,16]. Such studies on the inter-relations among enzyme activities, ECM fungi, and environmental variables help to elucidate the role of mycorrhizal fungi in sensitive environments (the topic of the current Special Issue), especially regarding nutrient cycling.

## 2. Materials and Methods

### 2.1. Description of Field Site and Field Sampling Methods

*P. taeda* roots were sampled across a transitional zone between an old field and second-growth forest at the Upper Sardis Wildlife Management Area in Lafayette County, Mississippi, USA (Lat: 34.481° N, Long: −89.589° W). The sampling site was composed of an open field vegetated with herbaceous vegetation and young *P. taeda* trees (<5 years old), near an abrupt transition into a mature second-growth forest. The open field was typical of a seasonally flooded plain that might retain excess water during wet seasons. Several days of precipitation occurred before sampling so that up to 5 cm of standing water was present in low areas on the day of collection. The second-growth forest was a mixed hardwood forest, including oaks (*Quercus* spp.) and hickories (*Carya* spp.) as co-dominant species in the canopy along with *P. taeda*.

On 15 January 2010, two transect lines (180 m long, 16 m apart) were established in an east-west orientation perpendicular to the field-forest boundary. Transects were established so that 85 m was in the forest and 95 m was in the field. Along each transect, rhizosphere soil was collected from under the nearest *P. taeda* tree, approximately every 20 m. Ten samples were taken along transect 1 while eight samples were collected along transect 2. An additional sample (sample 18) was added to obtain a sample from underneath a seedling that was located midway between the two transects. Trees sampled in the forest were binned by height as either seedlings (<0.2 m tall) or mature adults (>10 m tall); no saplings were found along the transects in the forest. Trees sampled in the field were all classified as saplings and were 1.12–2.81 m tall. Altogether, 19 individual tree rhizospheres were sampled over the two transects, representing ten saplings in the field, three seedlings in the forest, and six adult trees in the forest.

Beneath each tree, trowels were used to excavate ECM root segments. For trees <0.2 m tall, the entire plant was collected. Root samples from larger trees were collected by digging down at the base of the tree, identifying up to three primary roots, and excising a 0.15 m section from each root segment, along with surrounding soil from the O and A horizons. Litter in the forest primarily consisted of several centimeters of dead pine needles and hardwood leaves. Cylindrical soil cores (25 mm diameter, 150 mm deep) were collected underneath each tree to characterize soil properties at each sample location. Soil sand ECM root samples were brought back to the laboratory in insulated coolers, stored at 4 °C, and processed within 17 day of collection.

### 2.2. Initial Laboratory Processing of Root Samples

Roots were hand washed over a 2 mm sieve to remove rhizosphere soil. All roots within a sample were pooled and a random subset of roots selected for assessment. Using a dissecting microscope, the number of root tips with viable ECM colonization was counted, and each root tips was classified into a morphotype based on morphological distinctions such as color, texture, branching patterns, and emanating hyphae or rhizomorphs.

Two root tips per morphotype observed in each sample were removed for identification via DNA sequencing. While all morphotypes observed per sample were counted and saved for molecular identification, only dominant morphotypes (those with sufficient numbers of root tips for assays) were used for determination of enzymatic activity (typically 2–3 morphotypes per sample). Up to 27 root tips were collected and assayed for each of these dominant morphotypes: five tips for assays of each of phenol oxidase, peroxidase, β-glucosidase, and phosphatase, five tips for a sample control, and two tips for molecular identification of ECM type. Whenever possible, extra root tips were collected to allow replicate enzyme assays for the same morphotype to check for consistency. Tips collected for molecular analyses were stored at −20 °C.

### 2.3. Enzymatic Assay

We assayed the activity of the enzymes phenol oxidase, peroxidase, β-glucosidase, and phosphatase, as these groups of enzymes have previously been shown to be functionally important in ECM fungi (e.g., [15,16]). To determine potential activities of ECM associated enzymes, an assay was designed based on that of Courty *et al.* [17] but using colorimetric rather than fluorescent substrates. Because colorimetric assays involve longer reaction incubation times, we used different root tips from each sample to assay the activity each enzyme, rather than transferring the same root tip serially between the different artificial substrates for each assay. This approach assumed some amount of consistency in enzyme activities among different root tips of the same morphotype within a sample, which was supported by relatively low standard errors among replicate assays of the same enzyme across different root tips. A total of 156 tips (field: 94, forest: 62) were assayed for phenol oxidase activity, 150 tips (field: 96, forest: 54) were assayed for β-glucosidase activity, 148 tips (field: 94, forest: 54) were assayed for peroxidase activity, and 150 tips (field: 96, forest: 54) were assayed for phosphatase activity. The specific artificial substrate used for assays of phenol oxidase and peroxidase activity was 3,4-Dihydroxyphenylalanine (l-DOPA). Assays for phenol oxidases and peroxidase that use only a single model substrate are likely to only capture a fraction of the oxidative potential from environmental samples because it is susceptible to chemical oxidation by manganese and iron found in the soil [30]; however, alternative assays that use different substrates with similar redox potential as l-DOPA generate similar results as those with l-DOPA, suggesting our results are likely robust to this issue [30,31]. *p*-Nitrophenyl-linked substrates were used to assay β-glucosidase and phosphatase activity [32].

Substrates were prepared and assays ran following general procedures demonstrated by Jackson *et al.*, 2013 [33] with some modifications. Assays used 5 mM substrate solutions prepared in 50 mM of acetate buffer (pH 5.0). Individual assays contained 150 μL of root tip sample (three to five root tips in acetate buffer) and 150 μL of substrate solution, for a total of 300 μL. Sample controls contained the same amount of sample but 150 μL acetate buffer instead of substrate solution. Substrate controls consisted of 150 μL of acetate buffer and 150 μL substrate solution [33]. Assays and controls for peroxidase activity also contained 15 μL 0.3% hydrogen peroxide. All assays occurred in 96-well microplates and were run with experimentally determined optimum incubation time for assays of (phenol oxidase, peroxidase, and β-glucosidase activity: 4 h, phosphatase activity: 2 h). All plates were incubated on a Microplate Genie (Scientific Industries, Bohemia, NY, USA) at room temperature with periodic mixing (1000 rpm, 2–3 min) every 30 min of the incubation period to ensure that mycorrhizal tips had equal access to substrates. After the incubation period, microplates were centrifuged briefly.

Following incubation, the liquid in each assay was transferred to a new 96-well microplate for determination of absorbance using a BioTek Synergy microplate spectrophotometer (Biotek, Winooski, VT, USA). Assays and controls for phenol oxidase and peroxidase were read directly (at 460 nm), while assays for β-glucosidase and phosphatase received 20 μL 1 M NaOH to enhance the color of the end product prior to reading at 410 nm. Absorbance at the appropriate wavelength is directly related to the amount of substrate formed, which is dependent upon the amount of microbial enzymatic activity [32,34]. Following the completion of assays, the remaining ECM root tips in the reaction microplate were dried at 60 °C for 48 h and weighed to determine dry sample weight (g), so that potential enzyme activities could be expressed per gram of mycorrhizal dry weight. Although a proportion of this weight is made up of plant mass, mycorrhizal weight is approximately proportional to the surface area of root occupied by ECM fungi [7].

Final sample absorbance was calculated as the absorbance of the assay less the absorbance from the substrate control (reflecting abiotic substrate hydrolysis or oxidation). Sample control absorbance was consistently zero. Absorbance was related to enzyme activity following a standard curve that relates the concentration of end product to absorbance [32]. Because the outcome of the peroxidase assay depends on both phenol oxidase and peroxidase activity, final net peroxidase activity was determined by subtracting phenol oxidase activity from gross peroxidase activity. Final activity was expressed as µmol substrate consumed h^−1^·g·sample^−1^.

### 2.4. Molecular Characterization of ECM Species

Two ECM root tips from each morphotype from each sample were collected, frozen at −20 °C, and stored for approximately two months until DNA extraction, PCR, and sequencing were performed. DNA was extracted from root tips from each sample using components of a Sigma Extract-N-Amp extraction kit (Sigma-Aldrich, St. Louis, MO, USA). 10 µL of the Sigma Extraction Buffer was added to each root tip, which was heated to 65 °C for 10 min, 95 °C for 10 min, and then received 30 µL of Sigma Neutralization Solution and 60 μL PCR-grade water. Samples were immediately used in PCR or stored temporarily for several days at 4 °C prior to PCR.

To facilitate Sanger sequencing of ECM fungal species colonizing root samples, the Internal Transcribed Spacer (ITS) region of the fungal nuclear genome was amplified using the fungal-specific forward and reverse primers, NSI1 and NLB4 [35]. Amplification reactions for each sample consisted of 2.7 μL PCR-grade water, 4 μL of 2X RedTaq Premix (Apex Bioresearch Products, Inc., San Diego, CA, USA), 0.4 μL of each primer (10 μM stock concentration), and 0.5 μL of DNA extract for a total of 8 μL per reaction. Amplification occurred in sterile 96-well PCR plates which were sealed with a sterile silicone sealing mat, centrifuged briefly, and amplified under the following conditions: initial denaturation for 3 min at 94 °C; 30 cycles of denaturation for 45 s at 94 °C, annealing for 45 s at 58 °C and extension for 72 s per cycle at 72 °C; and a final extension of 10 min at 72 °C. Amplification success was checked on a 1% agarose gel with SYBR^®^ Safe DNA gel stain (Molecular Probes, Eugene, OR, USA). Excess primer and unincorporated nucleotides were removed enzymatically using ExoSAP-IT (USB Corporation, Cleveland, OH, USA) by adding 0.25 µL ExoSAP-IT and 4.75 µL sterile PCR-grade water to 5 µL of the PCR product. Reactions were incubated at 37 °C for 45 min, then 80 °C for 20 min, and finally 4 °C for at least 5 min.

Sequencing was performed using the forward primer ITS1F [36] and the Big Dye Terminator Sequencing Kit (v3.1, Invitrogen Corp., Grand Island, NY, USA). Each Big Dye reaction contained 0.4 µL Big Dye Reaction Premix, 1.8 µL Big Dye 5× sequencing buffer, 0.5 µL of the forward primer at 10 µM concentration, 6.3 µL of PCR-grade water, and 1 µL of the cleaned PCR product. Amplification conditions were 96 °C for 1 min; followed by 35 cycles of 95 °C for 30 s, 50 °C for 20 s, and 60 °C for 4 min. Reactions were dried and shipped overnight to the DNA Lab at Arizona State University, in Tempe, Arizona, where the Big Dye reactions were purified and read on an Applied Bioscience 3730 capillary genetic analyzer (Applied Biosystems, Foster City, CA, USA).

The fungal DNA sequences obtained were edited manually in Geneious software (Biomatter Ltd., Auckland, New Zealand), correcting ambiguous bases associated with dye blobs and elsewhere when possible. All sequences with >3% ambiguous bases or <200 base pairs long were deleted. Remaining sequences were subjected to operational taxonomic unit (OTU) assembly (at 97% similarity) using CAP3 software [37] on the University of Alaska, Fairbanks (UAF) informatics server, as described previously [38] using default settings except the following: maximum overhang percent length = 60, match score factor = 6, overlap percent identity cut-off = 96, clipping range = 6. Grouping homologous sequences that are >97% similar as a specific OTU is a conservative approach employed by previous studies [39,40,41] that assumes a 0.2%–1.2% error rate produced by PCR and unidirectional sequencing, as well as ~1.5% divergence of the ITS region that may occur within some species at small spatial scales [42]. Consensus fungal sequences from each OTU were checked using BLAST (nucleotide) searches on the International Nucleotide Sequence Database (INSD), the User-Friendly Nordic ITS Ectomycorrhizal (UNITE) database, and the curated fungal ITS database on the UAF Informatics Portal to obtain best matches for taxonomic affiliation of OTUs. The ultimate decision on the best match to a sequence was based on both similarity and length of the match (Table S1). Sequences >97% similar in composition to database sequences from named, cultured fungi were considered the same OTU (hereafter, “species”). Sequences with matches showing 94%–97% similarity to a database sequence with an assigned species epithet, or matching a sequence identified only to genus were assigned into the respective genus and assigned a number (e.g., *Russula* 1). Similarly, those matches in the database < 94%, but greater than 90% were assigned to the appropriate taxonomic family. Any matches < 90% similar to database sequences were left out of the analyses. If sequence matches among the three sequence repositories showed equal affinity or similarity to multiple genera within a family, priority was given to the vouchered specimens residing on the UNITE or curated fungal ITS databases. Any species known to be strictly non-mycorrhizal was eliminated from the data set.

### 2.5. Soil Characterization

Subsamples of each soil sample were sent to the University of Southern Mississippi for determination of total carbon (C) and nitrogen (N) by flash combustion in an elemental analyzer. Soil texture was also characterized for each soil sample (Lamotte soil texture test kit). Soil water content was determined as function of the ratio of the initial soil weight and soil weight after being dried in a drying oven at 65 °C for 72 h.

### 2.6. Statistical Analyses

Distributions of all four enzyme activities were highly right-skewed, so we applied a log-transformation to all four. Abundance of each EMF species were relativized by their species maximum abundance observed in the data set [43] in order to put all species abundances on the same relative scale. All analyses were done with R statistical software, Version 3.1.1 [44].

To assess variation in environmental factors, each environmental factor (percent silt, percent sand, percent clay, soil carbon, soil nitrogen, soil moisture) was subjected to a linear model using the *lm* function from the *stats* package [44]. Variation across an ecotone due to environmental variables was condensed into a single value for use in subsequent analyses using linear discriminant analysis (the *lda* function from the *MASS* package [45]). The importance of the canonical function in describing the environmental data was assessed using the *lm* function. To assess the sources of variation in the mycorrhizal community matrix due to the environmental linear discriminant and ecotone, we used permutational manova based on 1000 permutations and the Bray-Curtis method for calculating dissimilarity indices via the *adonis* function in the *vegan* package [46]. Visualizations for this analysis were created using NMDS plots and the Bray-Curtis method via the metaMDS function in the *vegan* package and the *ggplot2* package [46,47]. Species richness was computed between the two habitats using the *specnumber* and *diversity* functions of the *vegan* package. To assess variation in enzyme activity due to the ecotone, the activity of each enzyme (phenol oxidase, peroxidase, β-glucosidase, and phosphatase) was independently subjected to a linear model using the *lm* function. Analyses were repeated using tree diameter as a covariate, but results were not qualitatively different from when diameter was excluded; therefore, we present results without the covariate.

We used structural equation modeling (SEM) to evaluate the three way relationship among soil characteristics, ECM, and enzyme activities. Since traditional SEM assumes no underlying structure to the data (all variables are derived from a normal distribution and all observations are independent) it was not an option for our dataset which contains variables best described from the quasipoisson distribution. Consequently, we used piecewise SEM in which paths are estimated in individual models and then pieced together to construct the causal model. This allows us to properly model ECM abundance using distributions appropriate for count data while maintaining linear models for soil characteristics and enzyme activities. Separate linear models were created for each enzyme activity to explore enzyme activities as a function of soil characteristics. Separate linear models were also created for each enzyme activity to determine differences based on ECM species. All linear models were created using the *lm* function. ECM species abundance was modeled as a function of soil characteristics using generalized linear models with distributions appropriate to the data. Specifically, models evaluating ECM abundance of the fungal families Atheliaceae, Cenococcum, Cortinariaceace, Russulaceae, and Tremellaceae were fit with the quasipoisson distribution and the log link while models for the fungal family Thelephoraceae were fit with the quasi distribution and the identity link. All generalized linear models were created using the *glm* function from the *stats* package [44]. Using the above described models (Supplementary Material), path coefficients were estimated following the piecewise SEM procedure from the *piecewiseSEM* package [48].

In order to assess model fit, we used a directional separation (d-sep) test which determines whether any paths are missing from the model and whether the model would be improved with the inclusion of the missing path(s) [49]. Specifically, a d-sep test asks whether the causally independent paths are significant while statistically controlling for variables on which these paths are conditional. First, the d-sep test considers a basis set, *i.e.*, the *k* pairs of variables that are not directly connected with an arrow in the causal model. Next, the probability of independence *p_i_* is estimated based on the *p*-value associated with the missing paths. With this information, we can then calculate the maximum likelihood estimate for each model based on Fisher’s *C* statistic:

(1) $$C\;=\;-2\overset{k}{\underset{i\;=\;1}{\sum }}(\ln pi)$$

which follows a chi-squared distribution with *2k* degrees of freedom [50]. Therefore, if a chi-squared test is run on the *C* statistic and *p* < 0.05, then the model is not a good fit and one or more of the missing paths contains some useful information. For our models, it was important to include a linear model relating phosphatase and peroxidase activity in order to account for the underlying correlational structure of these enzymes and ensure a model which well represents the data (Table 1).

**Table 1:** Fisher’s *C* statistic and the associated *p*-value for each path model relating environmental characteristics, enzymes and fungal species (Figure 1 and Figure 2). Non-significant *C* values suggest that we can accept the proposed model. *K* is the total number of free parameters in a model. *AIC* is Akaike’s information criterion of model selection and *AICc* is the *AIC* value corrected for small sizes.

Model	*C*	*p*-Value	*K*	*AIC*	*AICc*
Field Site Only	9.104	0.993	87	183.1	−52.47
Forest Site Only	17.71	0.981	95	207.7	−14.73
Species Model Across Sites	7.755	0.902	77	161.8	−131.2
Species Model Field	9.211	0.817	77	163.2	−55.19
Species Model Forest	3.469	0.991	80	163.5	−29.96

Additionally, using the *C* value associated to each casual model we calculated both Akaike’s Information Criterion (AIC), as well as *AIC* corrected for small sizes, *AIC_c_* according to the formulas:

(2a) $$AIC_{c}\;=\;C+2K$$

(2b) $$AIC_{c}\;=\;C+2K\left[\frac{n}{\left(n-K-1\right)}\right]$$

where *K* is the total number of free parameters in the model and *n* is the sample size.

**Figure 1:**
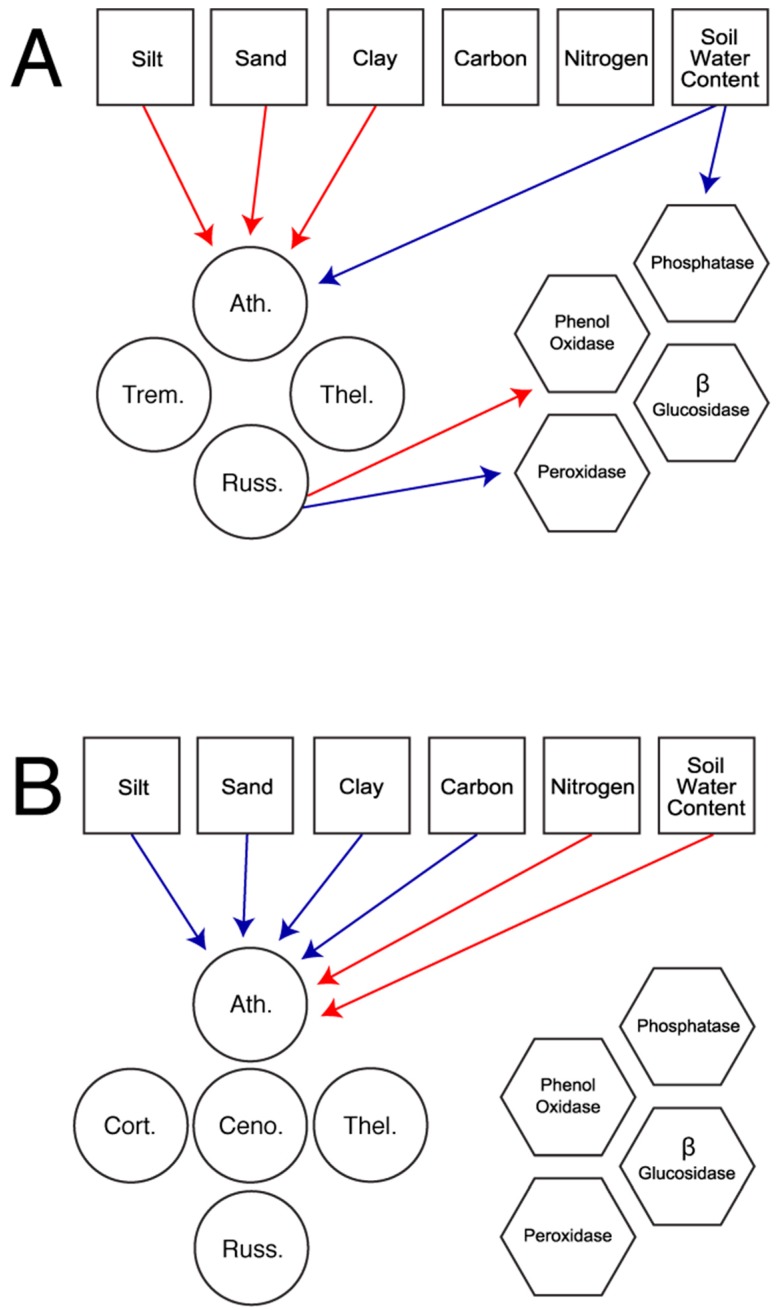
Most parsimonious piecewise structural equation model for full models of the Field (**A**) and Forest (**B**), exploring the effect of environmental variables (squares) on all present ectomyocrrhizal fungi (circles; Ath. = Atheliceae, Ceno. = Cenococcum, Cort. = Cortinariaceae, Russ. = Russulaceae, Thel. = Thelephoraceae, Trem. = Tremellaceae) or extracellular enzymes (hexagons). Enzymes could also be directly modified by environmental variables. Arrows indicate direction of effect. Only significant interaction pathways are represented. Red lines indicate positive interaction pathways while blue lines represent negative interaction pathways. Estimates, standard errors, and *p*-values associated with each pathway are found in Supplementary Tables S2–S4.

**Figure 2:**
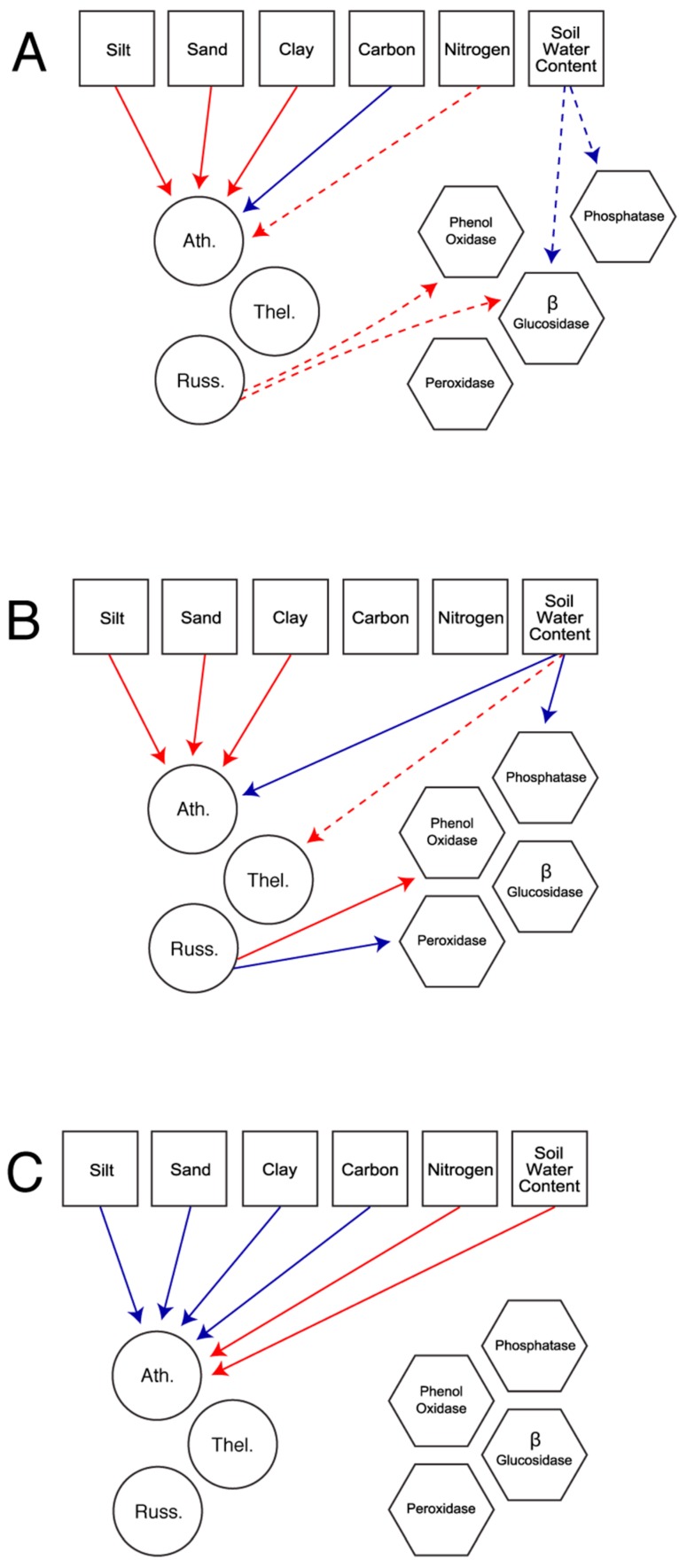
Most parsimonious piecewise structural equation model across sites (**A**); for the field only (**B**); and the forest only (**C**) exploring the effect of environmental variables (squares) on a subset of the ectomycorrhizal fungi community found across the ecotone (circles; Ath. = Atheliceae, Russ. = Russulaceae, Thel. = Thelephoraceae) or extracellular enzymes (hexagons). Enzymes could also be directly modified by environmental variables. Arrows indicate direction of effect. Only significant interaction pathways are represented. Solid lines indicate pathways significant at *p* < 0.05 while dashed lines indicate pathways significant at *p* < 0.1. Red lines indicate positive interaction pathways while blue lines represent negative interaction pathways. Estimates, standard error, and *p*-values associated with each pathway are found in Table 2 and Table 3.

In total, we constructed five models based on the fungal families present in each habitat. The first set of models considered all available fungal families in the field (Figure 1a) and the forest (Figure 1b). The second set of models explored patterns using only the three fungal families present in both habitats: Atheliaceae, Russulaceae, and Thelephoraceae. These models allow for the comparison of patterns across habitat (Figure 2a), in the field only (Figure 2b), and in the forest only (Figure 2c).

## 3. Results

### 3.1. Environmental Conditions

**Figure 3:**
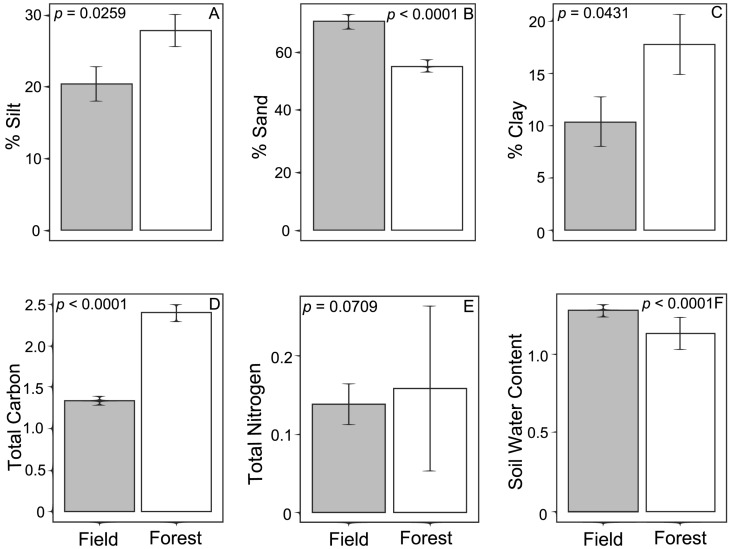
Soil characteristics for samples taken underneath loblolly pine (*Pinus taeda*) across an ecotone between forest and field habitats in north Mississippi, USA. Values represent the mean ± standard error for % silt (**A**), % sand (**B**), % clay (**C**), total Carbon (%) (**D**), total Nitrogen (%) (**E**) and soil water content (g water/g dry soil) (**F**) by the forest (white bars) and field (gray bars). Associated *p*-values are from linear models examining environmental characteristics as a function of location.

Linear models indicate the forest and field sites differed significantly in several environmental factors, except the amount of soil nitrogen (Figure 3). This variation was condensed into a single metric for use in subsequent analyses using linear discriminant analysis in which the first linear discriminate described greater than 99% of the variation between field and forest sites. Additionally, linear models assessing the importance of location in describing the canonical function generated from linear discriminant analysis were highly significant (*R*^2^ = 0.857, *p* < 0.0001).

### 3.2. ECM Community Structure

Both the field and the forest samples contained eight unique species but differed in the number of families represented. Field samples contained four families (Atheliaceae, Russulaceae, Thelephoraceae, Tremellaceae) and the forest contained five families (Atheliaceae, Gloniaceae (Cenococcum), Cortinariaceace, Russulaceae, Thelephoraceae). Shannon index values for the field (*H’* = 1.384) and the forest (*H’* = 1.503) indicate little difference in ECM community diversity between the two sites.

**Figure 4:**
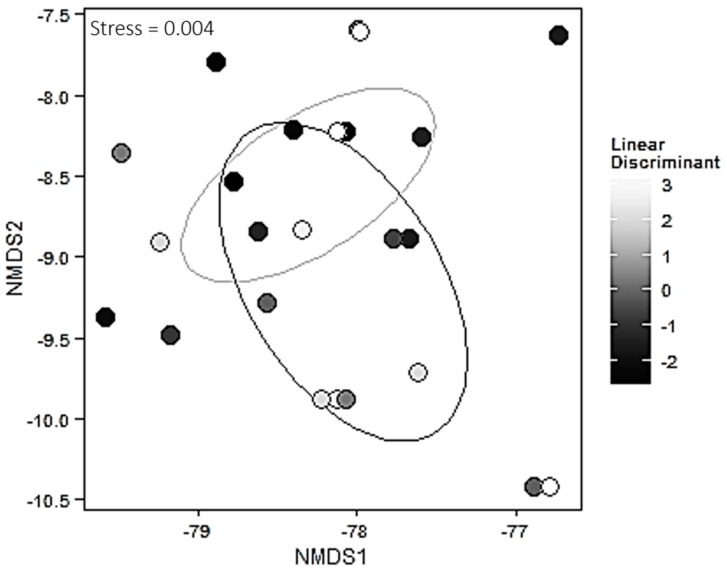
Nonmetric multidimensional scaling ordinations for ectomycorrhizal fungal communities as a function of the linear discriminant encompassing environmental variables (Percent Silt, Percent Sand, Percent Clay, Carbon, Nitrogen and Soil Water Content). Ellipses indicate 95% confidence intervals for field sites (gray) and forest sites (black).

Mycorrhizal community abundances were sorted into an ordination plot according to community similarity (Figure 4). Ecotone was a weak structuring factor of mycorrhizal assemblages (*R*^2^ = 0.06, *p* = 0.0643). The linear discriminant that encompasses the environmental factors (% sand, % silt, % clay, carbon, nitrogen, and soil water content) was a weak but significant structuring factor (*p* = 0.036) and explained 6.8% of the total variance from the community matrix (Figure 4). Two ECM species (OTUs) were significantly associated with one of the two habitats. Atheliaceae1 was strongly and significantly associated with the field (*p* = 0.005) and Cenococcum1 was strongly and significantly associated with the forest (*p* = 0.026).

### 3.3. Enzyme Activity

**Figure 5:**
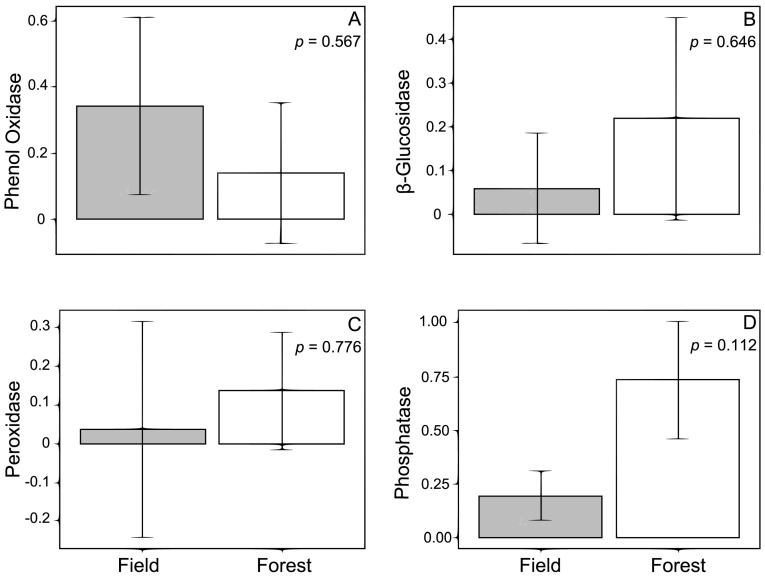
Activity of enzymes for samples taken underneath loblolly pine (*Pinus taeda*) across an ecotone between forest and field habitats in north Mississippi, USA. Values represent the mean ± standard error for phenol oxidase activity for a total of 156 tips (field: 94, forest: 62) (**A**); β-glucosidase activity for 150 tips (field: 96, forest: 54) (**B**); peroxidase activity for 148 tips (field: 94, forest: 54) (**C**); and phosphatase activity for 150 tips (field: 96, forest: 54) (**D**) for the forest (white bars) and field (gray bars). Enzymatic activity has the units (µ mol/h/g sample). Associated *p*-values are from linear models examining activity of enzymes as a function of location.

Linear models indicate that the forest and field sites did not significantly differ in the activities of the enzymes we measured (Figure 5): phenol oxidase (*p* = 0.567), peroxidase (*p* = 0.776), β-glucosidase (*p* = 0.646) and phosphatase (*p* = 0.112).

### 3.4. Piecewise SEM

Table 2 and Table 3 summarize the results for the most parsimonious piecewise structural equation model exploring the effect of environmental variables on extracellular enzyme activities or ECM fungi found in both the forest and field (Atheliceae, Russulaceae, Thelephoraceae) or across sites. Tables S2–S4 summarize the results for the most parsimonious piecewise structural equation model exploring the effect of environmental variables on extracellular enzymes or the full ECM fungal community at each site.

Piecewise structural equation modeling (SEM) revealed key differences in the way enzymatic activity is shaped by environmental characteristics and members of the ECM fungal community. While both the ECM fungal community and environmental characteristics were important contributors to enzyme activity in the field (Figure 1a and Figure 2b), this was not true in the forest (Figure 1b and Figure 2c). In the field, the presence of Russulaceae was associated with increased phenol oxidase activity (Full Field Model: *p* = 0.036, Species Field Model: *p* = 0.027) and decreased peroxidase activity (Full Field Model: *p* = 0.045, Species Field Model: *p* = 0.041). In terms of environmental conditions, soil water content was important and was associated with decreased phosphatase activity (Full Field Model: *p* = 0.048, Species Field Model: *p* = 0.048). These field-associated patterns also tended to hold in the full model, which disregarded habitat type to examine patterns for the fungal families present in both habitats (Atheliaceae, Russulaceae, and Thelephoraceae; Figure 2a). In this model, Russulaceae tended to be associated with an increase in the activity of phenol oxidase (*p* = 0.082) and β-glucosidase (*p* = 0.058), while soil water content tended to be associated with a decrease in the activity of β-glucosidase (*p* = 0.094) as well as phosphatase (*p* = 0.097).

Piecewise SEM also revealed key differences in the way Atheliaceae abundance is shaped via environmental characteristics, which were consistent for both the full within-habitat model and the reduced models. In the field, the measures of soil texture all had a positive correlation with Atheliaceae abundance (silt: *p* = 0.036, sand: *p* = 0.034, clay: *p* = 0.029, Figure 1a and Figure 2b), while soil water content had a negative relationship (*p* = 0.045, Figure 1a and Figure 2b). Neither soil carbon content (*p* = 0.355, Figure 1a and Figure 2b) or nitrogen (*p* = 0.647, Figure 1a and Figure 2b) showed a significant relationship to Atheliaceae abundance in the field site. These patterns were essentially reversed in the forest where the measures of soil texture all had negative effects on Atheliaceae abundance (silt: *p* < 0.001, sand: *p* < 0.001, clay: *p* < 0.001, Figure 1a and Figure 2c), as did soil carbon content (*p* < 0.001, Figure 1a and Figure 2c). However, soil water content (*p* < 0.001, Figure 1a and Figure 2c) and nitrogen content (*p* < 0.001, Figure 1a and Figure 2c) had a positive relationship with Atheliaceae abundance in the forest.

**Table 2:** Standardized total effects of environmental predictor variables on relative ECM fungal abundance across sites as derived from the models given in Figure 2. Silt, Sand, and Clay were log-transformed prior to analyses. Bold values indicate significance at *p* < 0.05.

		Atheliaceae			Russulaceae			Thelephoraceae		
		Estimate	Std. Error	*p* Value	Estimate	Std. Error	*p* Value	Estimate	Std. Error	*p* Value
Across Sites	Silt	**86.33**	**41.21**	**0.045**	3.885	34.9	0.912	−3.831	2.872	0.192
	Clay	**82.06**	**38.8**	**0.043**	6.224	33.69	0.855	−3.833	2.776	0.177
	Sand	**121.8**	**57.53**	**0.043**	6.243	45.35	0.891	−5.154	3.744	0.179
	Carbon	**−3.737**	**1.834**	**0.05**	0.816	0.772	0.299	0.064	0.091	0.487
	Nitrogen	46.71	24.26	0.064	−14.43	12.9	0.272	−0.785	1.22	0.525
	Soil Water Content	−3.559	3.787	0.355	2.543	4.543	0.58	0.466	0.423	0.279
Field	Silt	**129.2**	**56.32**	**0.036**	−72.37	64.68	0.280	−6.07	6.593	0.371
	Clay	**123.6**	**53.50**	**0.034**	−63.13	59.29	0.303	−5.734	6.098	0.361
	Sand	**180.7**	**75.47**	**0.029**	−97.33	86.07	0.275	−7.578	8.507	0.386
	Carbon	−2.745	2.884	0.355	0.735	5.492	0.895	0.829	0.619	0.199
	Nitrogen	16.43	35.21	0.647	−9.136	61.17	0.883	−7.614	7.714	0.338
	Soil Water Content	**−15.79**	**7.253**	**0.045**	−0.413	13.27	0.976	2.33	1.202	0.07
Forest	Silt	**−2361**	**207.7**	**<0.001**	−4483	1341598	0.997	−10.77	7.507	0.194
	Clay	**−3360**	**217.6**	**<0.001**	−4656	1396103	0.997	−11.58	7.855	0.184
	Sand	**−4282**	**275.6**	**<0.001**	−5707	1742072	0.997	−15.57	9.793	0.156
	Carbon	**−19.57**	**0.734**	**<0.001**	25.56	7098	0.997	−0.022	0.039	0.596
	Nitrogen	**244.7**	**13.11**	**<0.001**	36.51	45369	0.999	0.421	0.612	0.513
	Soil Water Content	**791.9**	**39.93**	**<0.001**	812.2	255192	0.998	1.505	1.499	0.349

**Table 3:** Standardized total effects of predictor variables on enzyme activities across sites as derived from the species models given in Figure 2. Silt, Sand, and Clay were log-transformed prior to analyses. The number of tips used to assess phenol oxidase activity were 53 for Atheliaceae (field: 48, forest: 5), 87 for Russulaceae (field: 31, forest: 56), and 21 for Thelephoraceae (field: 15, forest: 6). The number of tips used to assess peroxidase activity were 53 for Atheliaceae (field: 48, forest: 5), 79 for Russulaceae (field: 31, forest: 48), and 21 for Thelephoraceae (field: 15, forest: 6). The number of tips used to assess β Glucosidase and Phosphatase activity were 53 for Atheliaceae (field: 48, forest: 5), 81 for Russulaceae (field: 33, forest: 48), and 21 for Thelephoraceae (field: 15, forest: 6). Bold values indicate significance at *p* < 0.05.

			Phenol Oxidase			Peroxidase			β Glucosidase			Phosphatase		
			Estimate	Std. Error	*p* Value	Estimate	Std. Error	*p* Value	Estimate	Std. Error	*p* Value	Estimate	Std. Error	*p* Value
Across Sites	Environment	Silt	−20.12	15.55	0.206	17.39	14.99	0.255	0.085	15.53	0.996	−10.9	13.99	0.442
		Clay	−17.83	15.03	0.245	14.85	14.48	0.313	1.282	15.01	0.933	−11.42	13.52	0.405
		Sand	−22.52	20.28	0.276	18.61	19.54	0.348	0.342	20.25	0.987	−18.98	18.24	0.306
		Carbon	−0.079	0.492	0.874	−0.174	0.474	0.716	−0.204	0.491	0.681	−0.127	0.443	0.776
		Nitrogen	3.872	6.607	0.562	−2.781	6.366	0.665	−4.744	6.597	0.478	−3.381	5.943	0.574
		Soil Water Content	0.073	2.292	0.975	−1.262	2.209	0.572	−3.962	2.289	0.094	−3.528	2.062	0.097
	ECM	Atheliaceae	−0.267	0.695	0.703	0.233	0.708	0.744	−0.374	0.666	0.578	−1.158	0.698	0.107
		Russulaceae	1.261	0.704	0.082	−0.782	0.717	0.283	1.327	0.675	0.058	−0.284	0.708	0.691
		Thelephoraceae	−0.506	0.981	0.61	0.251	0.999	0.803	−0.584	0.94	0.564	−0.1	0.986	0.92
Field Site	Environment	Silt	−20.67	38.65	0.6	26.97	34.21	0.442	−6.476	22.89	0.781	12.08	17.74	0.506
		Clay	−18.23	35.75	0.617	23.71	31.65	0.465	−3.829	21.17	0.859	9.398	16.41	0.575
		Sand	−24.84	49.88	0.625	30.78	44.15	0.496	−10.64	29.54	0.723	10.66	22.89	0.648
		Carbon	−4.166	3.627	0.268	2.317	3.211	0.481	0.701	2.148	0.748	−1.459	1.655	0.394
		Nitrogen	48.66	42.23	0.298	−25.46	40.04	0.534	−10.71	26.79	0.695	6.003	20.76	0.776
		Soil Water Content	−3.916	7.05	0.586	2.205	6.241	0.728	−2.736	4.175	0.522	**−6.914**	**3.235**	**0.048**
	ECM	Atheliaceae	−0.173	0.921	0.853	−0.037	0.872	0.966	−0.344	0.625	0.589	−0.634	0.614	0.315
		Russulaceae	**2.658**	**1.105**	**0.027**	**−2.297**	**1.047**	**0.041**	1.059	0.751	0.175	0.139	0.738	0.852
		Thelephoraceae	−0.494	1.217	0.689	−0.006	1.154	0.996	−0.761	0.827	0.369	−0.712	0.813	0.392
Forest Site	Environment	Silt	6.743	43.22	0.880	−126.2	101.6	0.254	−150.9	206.4	0.489	−280.6	176.7	0.156
		Clay	8.022	45.22	0.864	−132.8	106.4	0.252	−155.9	216	0.494	−295.3	184.9	0.154
		Sand	10.07	56.38	0.863	−163.7	132.6	0.257	−188	269.2	0.508	−366.1	230.5	0.156
		Carbon	0.004	0.226	0.986	−0.052	0.532	0.924	0.66	1.079	0.560	0.593	0.924	0.542
		Nitrogen	4.254	3.52	0.266	1.633	8.279	0.849	−2.42	16.81	0.890	14.45	14.40	0.349
		Soil Water Content	−4.047	8.63	0.653	24.52	20.30	0.266	21.94	41.21	0.611	41.24	35.29	0.281
	ECM	Atheliaceae	0.997	1.221	0.433	−0.900	2.205	0.692	−1.883	3.897	0.639	−3.724	3.435	0.304
		Russulaceae	−0.364	0.440	0.427	0.976	0.795	0.248	1.684	1.405	0.258	−0.351	3.435	0.304
		Thelephoraceae	0.123	1.159	0.917	0.667	2.093	0.756	0.765	3.7	0.840	5.777	3.261	0.107

The full model, which disregarded ecotone, indicated that the measures of soil texture (silt: *p* = 0.045, sand: *p* = 0.043, clay: *p* = 0.043, Figure 2a) all had a positive correlation with Atheliaceae abundance. This model also indicated that soil carbon content had a negative effect on Atheliaceae abundance (*p* = 0.05, Figure 2a) while soil nitrogen content tended to have a positive effect (*p* = 0.064).

## 4. Discussion and Conclusion

Variation in the activity of enzymes associated with ECM fungi across an ecotone was complex in our study. While there were no overall patterns in enzyme activity in regards to the habitats, when the enzymatic activities of different individual ECM families are considered, our results reveal interesting patterns that emphasize the role that ECM family identity plays in shaping these interactions. Additionally, our results reveal the intricate role that environmental characteristics play in shaping both the ECM fungal community, as well as the activities of ECM associated enzymes.

We hypothesized that the activities of phenol oxidase and peroxidase would be higher in the forest than in the field because these enzymes contribute to the breakdown of lignin [30], a major component of wood, and woody debris was assumed to be more abundant in the forest. Individual statistical models considering enzyme activity solely as a function of habitat type revealed no significant differences in enzyme activity between the forest and field; however, when ECM family identity was taken into account, Russulaceae was associated with increased phenol oxidase activity and decreased peroxidase activity in the field, but not with enzyme activity in the forest. The ability of Russulaceae to alter phenol oxidase and peroxidase activity supports previous research with members *Russula* and *Lactarius* (both genera within the Russulaceae family, and also both present in our study) [51], but that research did not examine patterns in enzymatic activity, only whether those genera were active or not with regard to these enzymes. Our results enhance this work and indicate conflicting selection for enzymes that decompose lignin, possibly as a result of the evolutionary origins of genes associated with lignin peroxidase production. The ability to produce lignin peroxidase is a widespread trait for members of the Basidiomycota [30], which includes the Russulaceae, and 68% of the fungal taxa screened in one study possessed at least one gene associated with lignin peroxidase [52]. However, more recent work indicates that the genes associated with lignin peroxidase production in *Russula* and *Lactarius* may have different evolutionary origins than other members of the Basidiomycota [53]. Such evolutionary differences might explain why enzymes associated with lignin decomposition were most likely to be associated with fungi from the family Russulaceae, and why these enzymes responded differently across the ecotone. Furthermore, our results underline the importance of understanding the function of individual ECM families in describing enzyme production, especially in response to lignin degradation.

Activities of phosphatase and β-glucosidase did not vary across the ecotone or among morphotypes, suggesting no significant variation in carbohydrate or organic phosphate utilization. This result may be a reflection of the analyses performed and how the fungal community was categorized. In order to have enough statistical power to adequately examine these relationships, ECM fungi had to be grouped by family rather than by species. This left us with relatively few fungal families to examine, and species differences in enzyme production were therefore not discernible. Differences in habitat characteristics may have also led to this outcome. In mixed forests, which have both hardwood and pines present, β-glucosidase activity tends to be lower for soil collected from pine stands compared to that collected from around hardwoods [54], suggesting a sensitivity to the host plant rather than to specific ECM communities. Activities of extracellular enzymes associated with Scots pine (*Pinus sylvestris*) seedlings infected with either a fungal species classified as a litter decomposer (*Lepista nuda*) or one of two possible ECM fungal species (*Thelephora terrestris* or *Suillus bovinus*) found that both ECM fungi showed low lignocellulase activity, including low β-glucosidase activity [55]. Previous research examining phosphatase activity suggests that both ECM species and host species identity are important for describing variation in activity of that enzyme [56,57]. Thus, a number of factors could account for the lack of differences between forest and field habitats in terms of the activity of these enzymes, including the generally low diversity of ECM fungi in our samples.

While there were no significant differences in overall enzyme activity between forest and field habitats, to some extent environmental characteristics at each site could be related to ECM enzyme activity. Soil water content tended to decrease both β-glucosidase and phosphatase activity overall, but the effect of soil water content on phosphatase activity was only significant in the field habitat. Water availability likely alters enzyme activity via diffusion rates such that high diffusion rates (which are present in areas with higher water, as is suggested in our field site) decrease the rate of return on investment to the enzyme producer, resulting in down-regulation of enzyme production [58].

Given that abiotic variables establish the landscape in which enzyme producers compete, and that the forest and field differed significantly in many of their environmental characteristics, it is surprising we did not see more differences in enzyme activity related to the environmental characteristics. Indeed, previous research has demonstrated that extracellular enzyme activity can be influenced by various soil properties including total N [54,59], total C [59], and soil texture [60] as well as general environmental factors such as temperature, moisture, and pH [30,61]. Interestingly, oxidative (phenol oxidase and peroxidase) and hydrolase (β-glucosidase and phosphatase) activities in soils respond in opposite directions to such environmental factors, possibly due to the complexity of the molecules involved [30,31,62]. Abiotic controls, such as the type of substrate (soil texture) or general conditions (water availability, pH, temperature), can also shape extracellular enzyme activities via enzyme stabilization [33,63,64]. Thus, the fact that we did not see more patterns in enzymatic activity that could be linked to environmental characteristics indicates that controls on enzyme activity in the field may be more intricately linked to members of the ECM community.

In contrast to the field, activities of enzymes in the forest were not significantly influenced by any environmental characteristics. A number of environmental factors present in the forest but not present in the field may account for this lack of effect. For example, soil organic matter and climate have both been shown to strongly influence enzyme activity such that low soil organic matter and dry conditions may stabilize enzyme activity while wetter climate and high humus content may reduce enzyme activity [30,65]. Since the forest is likely to have higher soil organic matter then the field, it is probable that this played a role in obfuscating differences in enzyme activity between the two habitats and influences on enzyme activity within the forest habitat. Additionally, our study focused on four main enzymes known to be functionally important in ECM; however, the limited breadth of our enzyme activity profile may not have been able to capture the complexity of the enzyme profile produced by ECM in the different habitats (*sensu* [15,57]). For example, the forest litter layer may contain higher cellulosic content from leafy materials, necessitating an increase in the production of cellobiohydrolase (which we did not assay), indicating ECM types that are more associated with processing carbon sources as opposed to an N source (lignin-derived phenolics).

Activities of enzymes in the forest were also not significantly associated with any ECM species despite the fact that the two habitats also did not differ in ECM community structure in terms of either the number of species they contained or their diversities (Shannon diversity index, which takes into account both species numbers and relative abundances). Despite these similarities, there were more diverse ECM communities, in terms of families present, in the forest compared to the field. The lack of strong ECM control on potential enzyme activities indicates that any ECM specialization between the habitats did not translate to differences in the activity of enzymes. This observation may suggest that higher diversity of ECM fungi in the forest may result in partitioning of resources among ECM fungi and also a functional complementarity among species and within rhizospheres [15]. These results differ from previous findings which suggest that taxonomic diversity is highly correlated with functional diversity in ECM [66], possibly due to substantial differences in ECM morphology which contribute to the ability of the fungi to explore the substrate and transport nutrient to the host plant [23,57,67]. Interestingly, while these so-called exploration types have been linked to differences in enzyme activities, members our most active family Russulaceae appears to be unaffected by exploration type when it comes to explaining variation in enzyme activity, and instead are highly influenced by genetic distance between species within the family [57]. This suggests that the ability of an ECM fungal species to produce these enzymes has been selected for and conserved over evolutionary time. Because our limited sample set necessitated that ECM species be collapsed into families, it is possible that much of the variation in enzyme activity was also collapsed, limiting our ability to detect differences across the ecotone, but revealing broad scale patterns at the family level.

Some ECM species may be more suited to the forest and others more suited to the field, perhaps based on differences in environmental factors and competition among ECM species. Members of the family Atheliaceae were highly influenced by environmental characteristics. Specifically, in the field the proportions of silt, sand, and clay all increased Atheliaceae abundance, while soil water content decreased it. These relationships were essentially reversed in the forest where the abundance of Atheliaceae was negatively related to the proportion of silt, sand, and clay and positively related to soil water content and total nitrogen, suggesting that members of the family Atheliaceae are particularly vulnerable to alterations in their abiotic environment. Alternatively, the differential effects of forest and field could represent an artifact of binning our samples into families, and individual species with the Atheliaceae might respond differently and be found in the different habitats.

Previous research indicates congeneric ECM fungal species may differentially adapt their enzymatic capacities in different ecosystems [18,66]. To explore this idea, we examined relationships among environmental characteristics, ECM fungi and activity of enzymes for species that were found both in the field and the forest. Only three ECM families were present in both the field and the forest (Atheliaceae, Russulaceae, and Thelephoraceae). Members of the family Russulaceae showed the most enzymatic activity but this pattern was most prominent in the field site. Specifically, members of this family significantly increased phenol oxidase activity but decreased peroxidase activity, further emphasizing the complex nature of these relationships, and, in general, our observed variation in enzyme activity supports the idea that specific fungal groups may produce enzymes that target only certain subsets of nutrients available in the environment [67].

Our study is perhaps most limited by the confounding effect of tree age, which can influence both enzyme activity and ECM community [7,30,31]. The distribution of tree maturity levels between field and forest sites is disparate, with all of the field samples being saplings, compared to adults and seedlings from the forest site. Because of this confounding factor, analyses attempting to account for the disparity in tree age did not have sufficient power; however, when tree diameter was used as a covariate, analysis results did not differ from when it was excluded. This result indicates that tree age may not play a strong role in this system, but future studies designed to consider this possibility may elucidate stronger results.

This study is one of the first to examine differences in ECM fungal community composition and functionality patterns across a habitat boundary. It reveals key differences between the two habitats not only in ECM community composition but also in how the ECM community affects the activity of extracellular enzymes, emphasizing the complexity of these interactions. These results indicate that ECM fungi may have different foraging niches in the soil, corresponding to differences in their ability break down organic materials. Although there is still much to learn about host-symbiont interactions and the factors that dictate the distribution of ECM fungi and their enzymatic activity, our study makes important strides in understanding the importance of soil composition and environmental factors that may predict ECM fungal species community composition and functionality.

## Supplementary Materials

Supplementary File 1
