# Supplementary material for: Ectomycorrhizal Fungal Communities and Enzymatic Activities Vary across an Ecotone between a Forest and Field
Source: J Fungi (Basel). 2015 Aug 28;1(2):185–210. doi: 10.3390/jof1020185 (PMC5753110; doi:10.3390/jof1020185)
Supplement: Supplementary File 1 [file jof-01-00185-s001.docx]

**Supplementary Material**

Summary ECM OTUs found and their similarity to taxonomic database hits.

**Table S1A.** **Table of best UNITE matches.** Abbreviations indicate: “Score”—the BLAST score, E-value—the BLAST E-value, Prcnt—percent identity over the BLAST alignment, MisM—the number of mismatches over the BLAST alignment, Qstart—the position in the query sequence where the BLAST alignment starts, Qend—the position in the query sequence where the BLAST alignment ends, Rstart—the position in the reference sequence where the BLAST alignment starts, and Rend—the position in the reference sequence where the BLAST alignment ends.

| **OTU** | **UNITE Best Match** | **Score** | **E-value** | **Percent** | **MisM** | **Qstart** | **Qend** | **Rstart** | **Rend** |
| --- | --- | --- | --- | --- | --- | --- | --- | --- | --- |
| Atheliaceae1 | [EU649087 uncultured Amphinema](http://www.ncbi.nlm.nih.gov/entrez/query.fcgi?db=nucleotide&cmd=search&term=EU649087) | 963 | 0 | 91.07 | 37 | 17 | 730 | 18 | 731 |
| Cenococcum1 | [EU057125 uncultured Cenococcum](http://www.ncbi.nlm.nih.gov/entrez/query.fcgi?db=nucleotide&cmd=search&term=EU057125) | 1171 | 0 | 98.93 | 7 | 1 | 653 | 26 | 678 |
| Cortinariaceae1 | [EU821665 Cortinarius obtusus](http://www.ncbi.nlm.nih.gov/entrez/query.fcgi?db=nucleotide&cmd=search&term=EU821665) | 1109 | 0 | 94.94 | 24 | 1 | 709 | 715 | 13 |
| Cryptococcus1 | [GQ181171 Cryptococcus sp QMW 2009a](http://www.ncbi.nlm.nih.gov/entrez/query.fcgi?db=nucleotide&cmd=search&term=GQ181171) | 1347 | 0 | 99.2 | 5 | 1 | 747 | 46 | 793 |
| Lactarius1 | [EF685078 Lactarius miniatosporus](http://www.ncbi.nlm.nih.gov/entrez/query.fcgi?db=nucleotide&cmd=search&term=EF685078) | 1448 | 0 | 98.89 | 9 | 1 | 808 | 29 | 836 |
| Russula1 | [HQ604835 Russula cf pectinata 001114 28](http://www.ncbi.nlm.nih.gov/entrez/query.fcgi?db=nucleotide&cmd=search&term=HQ604835) | 1338 | 0 | 96.29 | 21 | 1 | 803 | 56 | 859 |
| Russulaceae1 | [UDB002670 Lactarius eucalypti](http://unite.ut.ee/bl_forw.php?nimi=UDB002670) | 1173 | 0 | 93.71 | 34 | 14 | 785 | 13 | 783 |
| Tomentella1 | [HM189969 Tomentella sp 2 BB 2010](http://www.ncbi.nlm.nih.gov/entrez/query.fcgi?db=nucleotide&cmd=search&term=HM189969) | 1090 | 0 | 97.5 | 14 | 1 | 639 | 67 | 703 |
| Tomentella2 | [UDB003336 Tomentella](http://unite.ut.ee/bl_forw.php?nimi=UDB003336) | 1136 | 0 | 95.49 | 28 | 17 | 724 | 1 | 706 |

**Table S1A.** **Table of top BLASTALL matches.** Abbreviations indicate: “HitName”—the BLAST match, “Hdesc”—description of the match, “Score”—the BLAST score, E-value—the BLAST E-value, Prcnt—percent identity over the BLAST alignment.

| **OTU** | **HitName** | **Hdesc** | **Score** | **E-Value** | **Ident** | **Prcnt** | **Hoverlap** |
| --- | --- | --- | --- | --- | --- | --- | --- |
| Atheliaceae1 | gi\|187403854\|gb\|EU649087.1\| | Uncultured ectomycorrhiza (Amphinema) clone Pine2_a1 18S ribosomal RNA gene, partial sequence; internal transcribed spacer 1, 5.8S ribosomal RNA gene, and internal transcribed spacer 2, complete sequence; and 28S ribosomal RNA gene, partial sequence | 468 | 0 | 537 | 95.72 | 560 |
| Cenococcum1 | gi\|156072277\|gb\|EU057125.1\| | Uncultured ectomycorrhiza (Cenococcum geophilum) clone ECUBC3 18S ribosomal RNA gene, partial sequence; internal transcribed spacer 1, 5.8S ribosomal RNA gene, and internal transcribed spacer 2, complete sequence; and 28S ribosomal RNA gene, partial seque | 627 | 0 | 646 | 98.93 | 653 |
| Cortinariaceae1 | gi\|194245018\|gb\|EU821665.1\| | Cortinarius obtusus voucher DAVFP 28814 18S ribosomal RNA gene, partial sequence; internal transcribed spacer 1, 5.8S ribosomal RNA gene, and internal transcribed spacer 2, complete sequence; and 25S ribosomal RNA gene, partial sequence | 553 | 0 | 673 | 94.92 | 703 |
| Cryptococcus1 | gi\|242134017\|gb\|GQ181170.1\| | Cryptococcus sp. QMW-2009a strain KTAPG1-11.63 18S ribosomal RNA gene, partial sequence; internal transcribed spacer 1, 5.8S ribosomal RNA gene, and internal transcribed spacer 2, complete sequence; and 26S ribosomal RNA gene, partial sequence | 720 | 0 | 742 | 99.2 | 748 |
| Lactarius1 | gi\|155212419\|gb\|EF685078.1\| | Lactarius miniatosporus isolate miniatosp 18S ribosomal RNA gene, partial sequence; internal transcribed spacer 1, 5.8S ribosomal RNA gene, and internal transcribed spacer 2, complete sequence; and 28S ribosomal RNA gene, partial sequence | 775 | 0 | 796 | 99 | 804 |

**Table S1A.** *Cont.*

| **OTU** | **HitName** | **Hdesc** | **Score** | **E-Value** | **Ident** | **Prcnt** | **Hoverlap** |
| --- | --- | --- | --- | --- | --- | --- | --- |
| Russula1 | gi\|315270819\|gb\|HQ604829.1\| | Russula cerolens isolate 001007-01 voucher UBC F18893 18S ribosomal RNA gene, partial sequence; internal transcribed spacer 1, 5.8S ribosomal RNA gene, and internal transcribed spacer 2, complete sequence; and 28S ribosomal RNA gene, partial sequence | 672 | 0 | 759 | 97.06 | 779 |
| Russulaceae1 | gi\|111219410\|gb\|DQ777991.1\| | Uncultured ectomycorrhiza (Russulaceae) internal transcribed spacer 1, partial sequence; 5.8S ribosomal RNA gene, complete sequence; and internal transcribed spacer 2, partial sequence | 523 | 0 | 657 | 93.99 | 694 |
| Tomentella1 | gi\|150035567\|gb\|EF619784.1\| | Uncultured Thelephoraceae clone 1S2.12.F04 18S ribosomal RNA gene, partial sequence; internal transcribed spacer 1, 5.8S ribosomal RNA gene, and internal transcribed spacer 2, complete sequence; and 28S ribosomal RNA gene, partial sequence | 597 | 0 | 600 | 99.83 | 601 |
| Tomentella2 | No Match Found | | | | | | |

**Table S2.** Standardized total effects of predictor variables on enzyme activities for the forest site as derived from the model given in Figure 4b. Silt, Sand, and Clay were log-transformed prior to analyses. The number of tips used to assess phenol oxidase activity were 5 for Atheliaceae, 28 for Cenococcum, 1 for Cortinariaceae, 56 for Russulaceae, and 6 for Thelephoraceae. The number of tips used to assess peroxidase activity were 5 for Atheliaceae, 28 for Cenococcum, 1 for Cortinariaceae, 48 for Russulaceae, and 6 for Thelephoraceae. The number of tips used to assess β Glucosidase and Phosphatase activity were 5 for Atheliaceae, 28 for Cenococcum, 1 for Cortinariaceae, 48 for Russulaceae, and 6 for Thelephoraceae. Bold values indicate significance at *p* < 0.05.

|  | **Phenol Oxidase** | | | **Peroxidase** | | | **β Glucosidase** | | | **Phosphatase** | | |
| --- | --- | --- | --- | --- | --- | --- | --- | --- | --- | --- | --- | --- |
|  | **Estimate** | **Std. Error** | ***p* Value** | **Estimate** | **Std. Error** | ***p* Value** | **Estimate** | **Std. Error** | ***p* Value** | **Estimate** | **Std. Error** | ***p* Value** |
| ***Environment*** |  |  |  |  |  |  |  |  |  |  |  |  |
| Silt | 6.743 | 43.22 | 0.88 | −126.2 | 101.6 | 0.254 | −150.9 | 206.4 | 0.489 | −280.5 | 176.7 | 0.156 |
| Clay | 8.022 | 45.22 | 0.864 | −132.8 | 106.4 | 0.252 | −155.9 | 215.9 | 0.494 | −295.3 | 184.9 | 0.154 |
| Sand | 10.07 | 56.38 | 0.863 | −163.7 | 132.6 | 0.257 | −188.0 | 269.2 | 0.508 | −366.1 | 230.5 | 0.156 |
| Carbon | 0.004 | 0.226 | 0.986 | −0.052 | 0.532 | 0.924 | 0.660 | 1.079 | 0.560 | 0.593 | 0.924 | 0.542 |
| Nitrogen | 4.254 | 3.52 | 0.266 | 1.633 | 8.279 | 0.849 | −2.420 | 16.81 | 0.89 | 14.44 | 14.40 | 0.349 |
| Soil Water Content | −4.047 | 8.63 | 0.653 | 24.52 | 20.30 | 0.266 | 21.94 | 41.21 | 0.611 | 41.24 | 35.29 | 0.281 |
| ***ECM*** |  |  |  |  |  |  |  |  |  |  |  |  |
| Atheliaceae | −0.014 | 1.271 | 0.991 | −0.326 | 2.700 | 0.907 | −2.244 | 4.836 | 0.655 | −3.522 | 4.234 | 0.430 |
| Cenococcum | −0.654 | 0.394 | 0.136 | 0.367 | 0.837 | 0.673 | −0.221 | 1.499 | 0.886 | 0.268 | 1.313 | 0.844 |
| Cortinariaceae | −0.668 | 0.465 | 0.189 | 0.391 | 0.989 | 0.703 | −0.279 | 1.771 | 0.879 | −0.313 | 1.550 | 0.845 |
| Russulaceae | −0.977 | 0.532 | 0.104 | 1.323 | 1.13 | 0.275 | 1.465 | 2.025 | 0.490 | −0.228 | 1.772 | 0.901 |
| Thelephoraceae | −1.085 | 1.272 | 0.418 | 1.353 | 2.703 | 0.630 | 0.333 | 4.841 | 0.947 | 6.019 | 4.238 | 0.193 |

**Table S3.** Standardized total effects of environmental predictor variables on relative ECM fungal abundance across sites as derived from the models given in Figure 4. Silt, Sand, and Clay were log-transformed prior to analyses. Tremellaceae was only present in Field sites while Cenococcum and Cortinariaceae were only present in the Forest. Bold values indicate significance at *p* < 0.05.

|  | | **Atheliaceae** | | | | **Cenococcum** | | | **Cortinariaceae** | | | **Russulaceae** | | | **Thelephoraceae** | | | **Tremellaceae** | | |
| --- | --- | --- | --- | --- | --- | --- | --- | --- | --- | --- | --- | --- | --- | --- | --- | --- | --- | --- | --- | --- |
|  |  | **Estimate** | **Std. Error** | **P value** |  | **Estimate** | **Std. Error** | **P value** | **Estimate** | **Std. Error** | **P value** | **Estimate** | **Std. Error** | **P value** | **Estimate** | **Std. Error** | **P value** | **Estimate** | **Std. Error** | **P value** |
| Field | Silt | **129.2** | **56.32** | **0.036** |  | - | - | - | - | - | - | −72.37 | 64.68 | 0.28 | −6.07 | 6.593 | 0.371 | −616.3 | 344402 | 0.999 |
|  | Clay | **123.6** | **53.50** | **0.034** |  | - | - | - | - | - | - | −63.13 | 59.29 | 0.303 | −5.734 | 6.098 | 0.361 | −565.3 | 321684 | 0.999 |
|  | Sand | **180.7** | **75.47** | **0.029** |  | - | - | - | - | - | - | −97.33 | 86.07 | 0.275 | −7.578 | 8.507 | 0.368 | −755.9 | 450164 | 0.999 |
|  | Carbon | −2.745 | 2.884 | 0.355 |  | - | - | - | - | - | - | −0.735 | 5.492 | 0.895 | 0.829 | 0.619 | 0.199 | −12.96 | 47681 | 1.000 |

**Table S3.** *Cont.*

|  | | **Atheliaceae** | | | | **Cenococcum** | | | **Cortinariaceae** | | | **Russulaceae** | | | **Thelephoraceae** | | | **Tremellaceae** | | |
| --- | --- | --- | --- | --- | --- | --- | --- | --- | --- | --- | --- | --- | --- | --- | --- | --- | --- | --- | --- | --- |
|  |  | **Estimate** | **Std. Error** | **P value** |  | **Estimate** | **Std. Error** | **P value** | **Estimate** | **Std. Error** | **P value** | **Estimate** | **Std. Error** | **P value** | **Estimate** | **Std. Error** | **P value** | **Estimate** | **Std. Error** | **P value** |
|  | Nitrogen | 16.43 | 35.21 | 0.647 |  | - | - | - | - | - | - | −9.136 | 61.17 | 0.883 | 7.614 | 7.714 | 0.338 | 87.54 | 729879 | 1.000 |
|  | Soil Water Content | **−15.79** | **7.253** | **0.045** |  | - | - | - | - | - | - | −0.413 | 13.27 | 0.976 | 2.33 | 1.202 | 0.07 | −6.146 | 81481 | 1.000 |
| Forest | Silt | **−3261** | **207.7** | **0.001** |  | 2704 | 798881 | 0.997 | −2372 | 3301890 | 0.999 | −4483 | 1341598 | 0.997 | −10.77 | 7.507 | 0.194 | - | - | - |
| Forest | Clay | **−3360** | **217.6** | **0.001** |  | 2931 | 868774 | 0.997 | −2462 | 3507505 | 0.999 | −4656 | 1396103 | 0.997 | −11.58 | 7.855 | 0.184 | - | - | - |

**Table S3.** *Cont.*

|  | | **Atheliaceae** | | | | **Cenococcum** | | | **Cortinariaceae** | | | **Russulaceae** | | | **Thelephoraceae** | | | **Tremellaceae** | | |
| --- | --- | --- | --- | --- | --- | --- | --- | --- | --- | --- | --- | --- | --- | --- | --- | --- | --- | --- | --- | --- |
|  |  | **Estimate** | **Std. Error** | **P value** |  | **Estimate** | **Std. Error** | **P value** | **Estimate** | **Std. Error** | **P value** | **Estimate** | **Std. Error** | **P value** | **Estimate** | **Std. Error** | **P value** | **Estimate** | **Std. Error** | **P value** |
|  | Sand | **−4282** | **275.6** | **0.001** |  | 3666 | 1085718 | 0.997 | −2994 | 4310772 | 0.999 | −5707 | 1742072 | 0.997 | −15.57 | 9.793 | 0.156 | - | - | - |
|  | Carbon | **−19.57** | **0.734** | **0.001** |  | 7.512 | 2293 | 0.997 | 0.673 | 15261 | 1.000 | 26.56 | 7098 | 0.997 | 0.421 | 0.612 | 0.513 | - | - | - |
|  | Nitrogen | **244.7** | **13.11** | **0.001** |  | −395.9 | 122950 | 0.998 | 65.29 | 268140 | 1.000 | 36.51 | 45370 | 0.999 | −0.022 | 0.039 | 0.596 | - | - | - |
|  | Soil Water Content | **791.9** | **39.94** | **0.001** |  | −555.9 | 165347 | 0.997 | 422.2 | 607694 | 0.999 | 812.2 | 255192 | 0.998 | 1.505 | 1.499 | 0.349 | - | - | - |

**Table S4.** Standardized total effects of predictor variables on enzyme activities in the field as derived from the full model given in Figure 4a. Silt, Sand, and Clay were log-transformed prior to analyses. The number of tips used to assess phenol oxidase and peroxidase activity were 48 for Atheliaceae, 31 for Russulaceae, 15 for Thelephoraceae, and 3 for Tremellaceae. The number of tips used to assess β Glucosidase and Phosphatase activity were 48 for Atheliaceae, 33 for Russulaceae, 15 for Thelephoraceae, and 3 for Tremellaceae. Bold values indicate significance at *p* < 0.05.

|  | **Phenol Oxidase** | | | **Peroxidase** | | | **β Glucosidase** | | | **Phosphatase** | | |
| --- | --- | --- | --- | --- | --- | --- | --- | --- | --- | --- | --- | --- |
|  | **Estimate** | **Std. Error** | ***p* Value** | **Estimate** | **Std. Error** | ***p* Value** | **Estimate** | **Std. Error** | ***p* Value** | **Estimate** | **Std. Error** | ***p* Value** |
| ***Environment*** |  |  |  |  |  |  |  |  |  |  |  |  |
| Silt | −20.67 | 38.65 | 0.6 | 26.97 | 34.21 | 0.442 | −6.476 | 22.89 | 0.781 | 12.08 | 17.74 | 0.506 |
| Clay | −18.23 | 35.75 | 0.617 | 23.71 | 31.65 | 0.465 | −3.829 | 21.17 | 0.859 | 9.398 | 16.41 | 0.575 |
| Sand | −24.84 | 49.88 | 0.625 | 30.78 | 44.15 | 0.496 | −10.64 | 29.54 | 0.723 | 10.66 | 22.89 | 0.648 |
| Carbon | −4.166 | 3.627 | 0.268 | 2.317 | 3.211 | 0.481 | 0.701 | 2.148 | 0.748 | −1.459 | 1.665 | 0.394 |
| Nitrogen | 48.66 | 45.23 | 0.298 | −25.46 | 40.04 | 0.534 | −10.71 | 26.79 | 0.695 | 6.003 | 20.76 | 0.776 |
| Soil Water Content | −3.916 | 7.05 | 0.568 | 2.205 | 6.241 | 0.728 | −2.736 | 4.175 | 0.522 | **−6.914** | **3.235** | **0.048** |
| ***ECM*** |  |  |  |  |  |  |  |  |  |  |  |  |
| Atheliaceae | −0.184 | 0.979 | 0.853 | −0.11 | 0.925 | 0.906 | −0.53 | 0.643 | 0.420 | −0.879 | 0.614 | 0.169 |
| Russulaceae | **2.646** | **1.167** | **0.036** | **−2.376** | **1.103** | **0.045** | 0.859 | 0.767 | 0.277 | −0.123 | 0.732 | 0.868 |
| Thelephoraceae | −0.503 | 1.266 | 0.696 | −0.05 | 1.196 | 0.967 | 0.905 | 0.831 | 0.291 | −0.900 | 0.794 | 0.271 |
| Tremellaceae | −0.057 | 1.247 | 0.964 | −0.361 | 1.179 | 0.763 | −0.923 | 0.819 | 0.275 | −1.212 | 0.782 | 0.139 |

**Code for Piecewise SEM Models**

*Field Only*

field.modlist = list(

lm(Act_phen2~logPropSilt + logPropSand + logPropClay + Carbon + Nitrogen + Soil.Water.Content, na.action=na.omit, data=BBT.field),

lm(Act_perox2~logPropSilt + logPropSand + logPropClay + Carbon + Nitrogen + Soil.Water.Content, na.action=na.omit, data=BBT.field),

lm(Act_Beta2~logPropSilt + logPropSand + logPropClay + Carbon + Nitrogen + Soil.Water.Content, na.action=na.omit, data=BBT.field),

lm(Act_Phos2~logPropSilt + logPropSand + logPropClay + Carbon + Nitrogen + Soil.Water.Content, na.action=na.omit, data=BBT.field),

glm(Atheliaceae~logPropSilt + logPropSand + logPropClay + Carbon + Nitrogen + Soil.Water.Content, family=quasipoisson(link="log"), na.action=na.omit, data=BBT.field),

glm(Russulaceae~logPropSilt + logPropSand + logPropClay + Carbon + Nitrogen + Soil.Water.Content, family=quasipoisson(link="log"), na.action=na.omit, data=BBT.field),

glm(Thelephoraceae~logPropSilt + logPropSand + logPropClay + Carbon + Nitrogen + Soil.Water.Content, family=quasi(link="identity"), na.action=na.omit, data=BBT.field),

glm(Tremellaceae~logPropSilt + logPropSand + logPropClay + Carbon + Nitrogen + Soil.Water.Content, family=quasipoisson(link="log"), na.action=na.omit, data=BBT.field),

lm(Act_phen2 ~Atheliaceae + Russulaceae + Thelephoraceae + Tremellaceae, na.action=na.omit, data=BBT.field),

lm(Act_perox2 ~Atheliaceae + Russulaceae + Thelephoraceae + Tremellaceae, na.action=na.omit, data=BBT.field),

lm(Act_Beta2 ~Atheliaceae + Russulaceae + Thelephoraceae + Tremellaceae, na.action=na.omit, data=BBT.field),

lm(Act_Phos2 ~Atheliaceae + Russulaceae + Thelephoraceae + Tremellaceae, na.action=na.omit, data=BBT.field),

lm(Act_phen2 ~ Act_perox2, na.action=na.omit, data=BBT.field))

*Forest Only*

forest.modlist = list(

lm(Act_phen2~logPropSilt + logPropSand + logPropClay + Carbon + Nitrogen + Soil.Water.Content, na.action=na.omit, data=BBT.forest),

lm(Act_perox2~logPropSilt + logPropSand + logPropClay + Carbon + Nitrogen + Soil.Water.Content, na.action=na.omit, data=BBT.forest),

lm(Act_Beta2~logPropSilt + logPropSand + logPropClay + Carbon + Nitrogen + Soil.Water.Content, na.action=na.omit, data=BBT.forest),

lm(Act_Phos2~logPropSilt + logPropSand + logPropClay + Carbon + Nitrogen + Soil.Water.Content, na.action=na.omit, data=BBT.forest),

glm(Atheliaceae~logPropSilt + logPropSand + logPropClay + Carbon + Nitrogen + Soil.Water.Content, family=quasipoisson(link="log"), na.action=na.omit, data=BBT.forest),

glm(Cenococcum~logPropSilt + logPropSand + logPropClay + Carbon + Nitrogen + Soil.Water.Content, family=quasipoisson(link="log"), na.action=na.omit, data=BBT.forest),

glm(Cortinariaceae~logPropSilt + logPropSand + logPropClay + Carbon + Nitrogen + Soil.Water.Content, family=quasipoisson(link="log"), na.action=na.omit, data=BBT.forest),

glm(Russulaceae~logPropSilt + logPropSand + logPropClay + Carbon + Nitrogen + Soil.Water.Content, family=quasipoisson(link="log"), na.action=na.omit, data=BBT.forest),

glm(Thelephoraceae~logPropSilt + logPropSand + logPropClay + Carbon + Nitrogen + Soil.Water.Content, family=quasi(link="identity"), na.action=na.omit, data=BBT.forest),

lm(Act_phen2 ~Atheliaceae + Cenococcum + Cortinariaceae + Russulaceae + Thelephoraceae, na.action=na.omit, data=BBT.forest),

lm(Act_perox2 ~Atheliaceae + Cenococcum + Cortinariaceae + Russulaceae + Thelephoraceae, na.action=na.omit, data=BBT.forest),

lm(Act_Beta2 ~Atheliaceae + Cenococcum + Cortinariaceae + Russulaceae + Thelephoraceae, na.action=na.omit, data=BBT.forest),

lm(Act_Phos2 ~Atheliaceae + Cenococcum + Cortinariaceae + Russulaceae + Thelephoraceae, na.action=na.omit, data=BBT.forest),

lm(Act_perox2 ~Act_Phos2, na.action=na.omit, data=BBT.forest))

*Across Sites*

test4.modlist = list(

lm(Act_phen2~logPropSilt + logPropSand + logPropClay + Carbon + Nitrogen + Soil.Water.Content, na.action=na.omit, data=BBT),

lm(Act_perox2~logPropSilt + logPropSand + logPropClay + Carbon + Nitrogen + Soil.Water.Content, na.action=na.omit, data=BBT),

lm(Act_Beta2~logPropSilt + logPropSand + logPropClay + Carbon + Nitrogen + Soil.Water.Content, na.action=na.omit, data=BBT),

lm(Act_Phos2~logPropSilt + logPropSand + logPropClay + Carbon + Nitrogen + Soil.Water.Content, na.action=na.omit, data=BBT),

glm(Atheliaceae~logPropSilt + logPropSand + logPropClay + Carbon + Nitrogen + Soil.Water.Content, family=quasipoisson(link="log"), na.action=na.omit, data=BBT),

glm(Russulaceae~logPropSilt + logPropSand + logPropClay + Carbon + Nitrogen + Soil.Water.Content, family=quasipoisson(link="log"), na.action=na.omit, data=BBT),

glm(Thelephoraceae~logPropSilt + logPropSand + logPropClay + Carbon + Nitrogen + Soil.Water.Content, family=quasi(link="identity"), na.action=na.omit, data=BBT),

lm(Act_phen2 ~ Atheliaceae + Russulaceae + Thelephoraceae, na.action=na.omit, data=BBT),

lm(Act_perox2 ~ Atheliaceae + Russulaceae + Thelephoraceae, na.action=na.omit, data=BBT),

lm(Act_Beta2 ~ Atheliaceae + Russulaceae + Thelephoraceae, na.action=na.omit, data=BBT),

lm(Act_Phos2 ~ Atheliaceae + Russulaceae + Thelephoraceae, na.action=na.omit, data=BBT),

lm(Act_phen2~Act_perox2 + Act_Beta2, na.action=na.omit, data=BBT.forest),

lm(Act_Beta2~Act_perox2, na.action=na.omit,data=BBT))

© 2015 by the authors; licensee MDPI, Basel, Switzerland. This article is an open access article distributed under the terms and conditions of the Creative Commons Attribution license (http://creativecommons.org/licenses/by/4.0/).
